# Editorial: Social Interaction in Neuropsychiatry

**DOI:** 10.3389/fpsyt.2021.683158

**Published:** 2021-04-29

**Authors:** Victoria Leong, Danilo Bzdok, Frieder M. Paulus, Kevin Pelphrey, Elizabeth Redcay, Leonhard Schilbach

**Affiliations:** ^1^Division of Psychology, Nanyang Technological University, Singapore, Singapore; ^2^Department of Psychology, University of Cambridge, Cambridge, United Kingdom; ^3^Mila - Quebec Artificial Intelligence Institute, Montreal, QC, Canada; ^4^Faculty of Medicine, Montreal Neurological Institute, McGill University, Montreal, QC, Canada; ^5^Social Neuroscience Lab at the Translational Psychiatry Unit, Department of Psychiatry and Psychotherapy, Lübeck University, Lübeck, Germany; ^6^Department of Neurology, University of Virginia, Charlottesville, VA, United States; ^7^Department of Psychology, University of Maryland, College Park, MD, United States; ^8^Medical Faculty, Ludwig Maximilians University, Munich, Germany

**Keywords:** social interaction, neuropsychiatry, two-person neuroscience, clinical disorders, autism spectrum disorder

## Introduction

Psychiatric disorders can affect and are even conditioned on our ability to successfully and enjoyably interact with other people. Conversely, facing difficulties in social relations or being socially isolated is known to increase the risk of developing a psychiatric disorder and strongly impacts symptom progression and health outcomes. This tight link between social interaction and risk for mental health challenges has been taken to suggest that psychiatric disorders can be construed as disorders of social interaction (1). This link places a focus on the dynamics and mechanisms of social interaction, which may usher in new research perspectives for quantitative, multi-scale approaches that aim to advance the transdiagnostic investigation of the behavioral and neural mechanisms of psychiatric disorders (2).

This Research Topic on “Social Interaction in Neuropsychiatry” attracted a sizable number of contributions from ~100 authors who addressed important questions about the nature of social interaction, its behavioral and neural mechanisms and relationship to psychiatric disorders. A total of 16 articles were published under this Topic, encompassed under four broad themes spanning clinical implications, developmental perspectives, contextual considerations on processing and methodological innovations.

## Clinical Implications Of Social Processing Gone Awry

A key theme of many articles in this Research Topic pertains to how disruptions in or atypical social processing can contribute to risk for neuropsychiatric disorders. At the same time, working to address social processing impairments can offer uncharted opportunities for resilience in the face of neuropsychiatric conditions. Blanchard et al. examined the ways in which sleep problems, commonly found in individuals with psychosis, lead to severe social impairments. Across clinical diagnoses, they evaluated multiple social domains, and found that addressing sleep problems can have a strong beneficial effect for improving both social skills and, indirectly, improving psychotic symptoms. This insight suggests a new avenue for approaching major neuropsychiatric symptoms, with an intervention that is relatively simple and without significant risk (e.g., a behavioral sleep intervention). Looking beyond the individual to the developmental dyad of mothers and their children, Apter-Levy et al. demonstrated that chronic depression alters mothers' dehydroepiandrosterone (DHEA) and DHEA-to-cortisol ratio. In turn, these alterations in hormonal levels drive reductions in sensitive maternal caregiving. However, as the authors point out, this is a delicate developmental “dance,” with improvements in maternal sensitivity eliciting from the child behaviors that further improve DHEA levels and balance DHEA-to-cortisol ratios, thus potentially initiating a positive social biofeedback mechanism. In a time at which many are carefully examining their own implicit biases towards others,  Hauschild et al. provide evidence for an own-age bias in facial emotion recognition for adolescents with and without autism spectrum disorder (ASD). This discovery has probably not been anticipated because in many fields autistic individuals would not have been predicted to be as susceptible to these common cognitive-perceptual biases. This is an intriguing new lead that deserves further study to understand the possible clinical implications. Finally, several systematic reviews appear in this research topic covering a range of both relatively mature, yet still exciting and growing areas of research. These include the role of speech prosody in psychopathology and linguistics (Lucarini et al.) and empathic accuracy in clinical populations (Jansen et al.) to relatively new areas including the study of social cognition in obsessive-compulsive disorder (Rum and Perry et al.).

## Developmental Perspectives On Disordered Social Interactions

Taking a developmental perspective, a trio of articles provide unique insights into how social interactive experiences during early childhood and adolescent years (whether experienced in person or in the virtual sphere) may have important and lasting consequences for lifelong mental health. In their article “A Social Neuroscience Approach to Interpersonal Interaction in the Context of Disruption and Disorganization of Attachment,” White et al. provide a unique account of the neurobiological “embedding” of disordered social interactions, and how this may lay a path toward psychopathology in later life. They describe a functional neuro-anatomical model of typical and disordered human attachment (NAMA) which explains the emergence of a disorganized attachment style through either hyper- or hypo-arousing social interactions with caregivers, who act as either a threatening or insufficient source of co-regulation, respectively.

Furthering the developmental theme, Cataldo et al. address a timely issue of “Social Media Usage and the Development of Psychiatric Disorders in Childhood and Adolescence,” a topic that has gained particular relevance in the aftermath of the global lockdowns imposed during the COVID pandemic. Problematic social media use during the ages of 10 to 19 is shown to be linked to a variety of mental health issues including depression, anxiety, eating, and neurodevelopmental disorders. Finally, Blair et al. provide empirical evidence that sexual abuse during the adolescent years is associated with a heightened neurobiological response to threatening stimuli – including both faces and animals. This heightened neural responsiveness was observed in regions beyond the amygdala, including the frontal gyrus and posterior cingulate gyrus, suggestive of widespread and fundamental changes in the individual's basic perceptual and emotional processing. Collectively, these articles highlight the developmental sequelae of disordered social interactive experiences during the formative years, and their impact on the circuitry and organization of the developing brain.

## Importance Of Context On Neural And Perceptual Processing

During real-world social interactions communicative information is embedded within a rich context that is complex, dynamic and often not directly observable. Social and nonsocial features of this complex environment interact to affect attention and neural processing. Further, distractors compete with the relevant social-communicative signals preventing effective social interaction. Thus, to understand both typical and atypical social interaction, social processes should be situated within the appropriate context. Two papers within this issue examine how context (e.g., presence of a person or environmental noise) affects neural processing and how that neural processing is closely related to social ability. Hernandez et al. examine the neural correlates of speech perception in the presence of ecologically-valid environmental noises in youth with and without autism spectrum disorder (ASD). Their findings suggest that a left-hemisphere language processing region may provide a compensatory mechanism in autism to attend to speech in the presence of competing background noise and potentially facilitate more successful social interactions.  Rolison et al. examine contextual effects on neural processing through a dual-brain EEG set-up. They demonstrate that the “resting brain” is different in the presence of a person, regardless of interaction status. Further, the extent to which one's social partner's physical orientation (i.e., face-to-face or back-to-back) modulated EEG gamma band power was related to self-reported social functioning. These findings underscore the importance of understanding the brain's “default mode” within the social context. The relatively novel methods discussed in this special issue also allow for a better understanding of real-world social perception.  Vettori et al. use a frequency tagging method to identify visual and neural preference for social (faces) compared to non-social (houses) stimuli presented within the same visual stream. By means of this method, the authors revealed a social bias in typically developing participants but no such bias in autistic participants.

## Methodological Innovations In Studying Social Interactive Processes

Understanding dysfunctional dynamics and mechanisms of social interaction requires methodological innovation at different levels that address experimental design and the overarching technical challenge of multi-subject measurement. A set of articles within this Research Topic highlight these important methodological challenges. Like faces, point-light walkers are frequently used as a measure of social perception. However, typical paradigms rely on a single person performing a non-communicative action (e.g., walking or biking). Okruszek and Chrustowicz et al. provide a novel open-access database of point-light displays that focus on reciprocal actions of multiple agents which incorporates social interactions, emotions, and differing perspectives (i.e., second-person and third-person). This toolbox addresses the need for obtaining controlled stimuli depicting social gestures and bodily interactions with vast potential for application in neuropsychiatric research. Also, challenging the experimental designs in social isolation, the study by Rolison et al. implies that the mere presence of someone in the extrapersonal space may shape neural oscillations, suggesting that neural activity is tuned towards the presence of others.

In their review Pan and Cheng et al. provide an overview of current approaches to examine two-person interactions and discuss advances and challenges in moving the field towards more interactive settings. They distinguish eye-to-eye contact, body-to-body synchronization, and brain-to-brain coupling as central dimensions to summarize recent findings across clinical diagnoses outlining a novel perspective for two-person approaches in psychological interventions of psychiatric disorders. Addressing specific challenges of examining body-to-body interactions in the fMRI, Renvall et al., provide a proof-of-concept for measuring two interacting subjects within one MRI scanner. Their custom built fMRI coil allowed participants to lie face-to-face in a shared peripersonal space. The sufficient signal properties and the feasibility of this setup provide a perspective for more accessible means to characterize neural mechanisms of emerging phenomena during social interaction. A comparable technical challenge is to characterize dynamic, coordinated behavior under controlled and reproducible conditions, specifically in children. Here,  Baillin et al. use the “Human Dynamic Clamp” [HDC, (3)] to characterize elementary forms of social behavior or higher-level phenomena such as intention attribution to distinguish children with autism spectrum disorder from typical developing controls during interpersonal coordination based on their objective behavioral profile.

## Conclusion

Taken together, this unique collection of articles clearly demonstrates that social neuroscience and related fields of research have not only taken an “interactive turn” by focusing on the social interactive nature of human behavior (4), but that this line of investigation is now being extended into the clinical domain of psychology and psychiatry. Here, it is being increasingly recognized that a mechanistic and meaningful understanding of psychiatric disorders can only be achieved by aiming for an integrative and pluralist account of psychopathology, which focuses on the dynamics of social interaction. Such an account will help to explain how social interactions—or their absence—can constitute a risk factor for the development of psychiatric disorders, and how helping patients to increase their social interaction skills may turn out to be a helpful transdiagnostic approach that promotes resilience and positively affects mental health. Finally, taking social interaction seriously may help to investigate how similarities across interaction partners affect quality of life (5) and lead to the refinement of patient-oriented approaches that are based on a deeper understanding of how interpersonal dissimilarities and mismatch in social interaction affect well-being. This could then be described as an inter-personalized, rather than a personalized psychiatry.

## Author Contributions

All authors listed have made a substantial, direct and intellectual contribution to the work, and approved it for publication.

## Conflict of Interest

The authors declare that the research was conducted in the absence of any commercial or financial relationships that could be construed as a potential conflict of interest.
